# The impact of emotional stimuli on response inhibition in an inpatient and day-hospital patient psychosomatic cohort

**DOI:** 10.3389/fpsyt.2023.1176721

**Published:** 2023-06-30

**Authors:** Sina Westbomke, Kathrin Schag, Birgit Derntl, Stephan Zipfel, Andreas Stengel

**Affiliations:** ^1^Department of Psychosomatic Medicine and Psychotherapy, University Hospital, Tübingen, Germany; ^2^Department of Psychiatry and Psychotherapy, Tübingen Center for Mental Health, University Hospital, Tübingen, Germany; ^3^Clinic for Psychosomatic Medicine, Charité Center for Internal Medicine and Dermatology, Humboldt-Universität zu Berlin and Berlin Institute of Health, Charite - Universitätsmedizin Berlin, Corporate Member of Freie Universität Berlin, Berlin, Germany

**Keywords:** emotional stop-signal task, psychosomatic, psychotherapy, response inhibition, stop-signal reaction time, day-hospital, inpatient, day-hospital patient

## Abstract

**Objectives:**

To correctly recognize and respond to your counterpart's emotion is essential for a successful get-together. To ensure this, emotional processes and inhibitory control are linked and interact with each other. However, this interaction can be altered in several mental disorders. In a group of psychosomatic patients, we investigated possible differences in the response inhibition between neutral and emotional stimuli and whether a psychosomatic inpatient and day-hospital patient treatment influences response inhibition profiles.

**Methods:**

One hundred and one patients, diagnosed with different psychiatric diagnoses (77 women, 41.43 ± 13.13 years), completed an emotional stop-signal task (ESST) and an impulsive behavior scale upon admission in an inpatient and day-hospital patient treatment on a psychosomatic ward (T0) and at discharge (T1). Patients with depressive disorders completed the test again after 1 year (follow-up measurement T2, *n* = 22). Emotional stimuli were angry and neutral faces. Stop-signal reaction time (SSRT) and stop-signal delay (SSD) were calculated as the main behavioral parameters.

**Results:**

We found a significantly higher SSRT for neutral than angry faces at both admission (8.538 ms, *p* < 0.001) and discharge (11.142 ms, *p* < 0.001), with a matching higher SSD for angry than neutral faces at both timepoints (T0: 8.360 ms, *p* < 0.001, T1: (6.950 ms, *p* < 0.001). The SSRT for angry faces significantly decreased after treatment (-8.485 ms, *p* = 0.0110). For neutral faces, the decrease failed to reach significance (−5.881 ms, *p* = 0.250). A significant decrease in SSRT for neutral faces in patients with depressive disorders was found 1 year after discharge compared with admission (−19.040 ms, *p* = 0.0380).

**Conclusion:**

Our data demonstrate a decreased response inhibition for neutral compared with emotional stimuli and an improved response inhibition for angry faces after discharge in a psychosomatic inpatient and day-hospital patient cohort. Additionally, patients with depressive disorders displayed a significantly better response inhibition for neutral faces 1 year after discharge compared with the baseline measurement. With this study, we provide more evidence for altered emotional response inhibition in different mental disorders and a hint that psychosomatic inpatient and day-hospital patient treatment may help to normalize it, even if the effects remained small and it needs further research to prove causality.

## 1. Introduction

Recognizing and reacting correctly to a counterpart's emotions are often essential for a successful interaction. To ensure this, emotion and inhibitory control are linked and affect each other (1). Several studies reported a significant effect of emotion on inhibitory control, although discordant results were reported on the effects (improvement/impairment) of the different emotions on inhibitory control. While some studies showed an increment of reaction time (RT) for emotional stimuli (2–4), others reported a decrease (5–8).

Fewer studies were performed focusing on a special part of inhibitory control, the response inhibition, which describes the ability to stop a prompted motor response due to an upcoming change of conditions, e.g., in a laboratory setting, usually an appearing stop-signal (9). Stop-signal tasks are established tests to examine response inhibition (9–13). In this task, the probands have the assignment to react to a stimulus—usually visual—fast and accurately, but not if a stop-signal (audio or visual) appears. This task is based on the assumptions of the horse race model (14, 16–19). In this model, the ongoing process of the original task (the go task) competes with the appearing process of the stop task. When the processing of the stop task is faster than the processing of the go task, the reaction is successfully inhibited. But in case the stop-signal appears late in the processing of the go-signal, this process is finished before the concurring process of the stop-signal and the reaction is executed despite the stop-signal. With this task, it is possible to estimate the latency of the stop process, the so-called stop-signal reaction time (SSRT) (10, 12–15). Alterations in response inhibition have been shown in participants diagnosed with different mental disorders, such as autism and attention deficit hyperactivity disorder (ADHD) (12, 16, 17), schizophrenia (17–23), obsessive-compulsive disorders (OCD) (12, 17, 24), depression (25–27), and eating disorders (28) compared with healthy controls.

To examine the impact of emotion on response inhibition, several studies combined a classical stop-signal task with emotional stimuli, which are often called an emotional stop-signal task (ESST). In healthy populations, most of the studies showed a decrease in response inhibition, represented in a slower SSRT for emotional stimuli compared with neutral ones, both when the pictures, but not the emotion, were task-relevant (29, 30) and when neither the pictures nor the emotion was task-relevant (1, 31, 32). A common explanation for this phenomenon is that emotional pictures draw attention, which is an important evolutionary advantage as one is able to quickly respond to an upcoming danger (1, 32, 33).

Only a few studies have used emotional stop-signal tasks with individuals diagnosed with mental disorders. Two studies with patients diagnosed with schizophrenia show a different result compared with the studies on healthy participants: Both studies showed facilitated response inhibition, i.e., faster SSRT, for the emotional, i.e., angry, stimuli in comparison with neutral stimuli (19, 23). These findings are contrary to the results of most studies with healthy participants, that most frequently report slower SSRT for emotional than neutral stimuli (1, 29–32). One explanation may be an altered processing of neutral faces in schizophrenia with problems interpreting them resulting in more distraction than easier-to-read emotional pictures (19, 34–37). One study with subclinically depressed individuals found slower SSRT for participants with subclinical depression compared with healthy controls in an ESST, when the emotion was not task-relevant, regardless of whether the presented pictures were neutral or emotional (in this case sad faces were used as emotional stimuli). When the emotion was task-relevant, they could not find any difference in SSRT or SSD (38). Another study not only investigated neutral and negative stimuli, but also positive stimuli. They found no differences in SSRT for patients with depressive disorders and healthy controls but an enhanced reduction of event-related potentials for positive stimuli compared with the controls (39). Similar results were shown for patients diagnosed with borderline personality disorder: no difference in SSRT, but slower event-related potentials regarding the early attention and stimulus evaluation showing an inhibition control deficit for emotional stimuli (40).

Regarding emotion recognition, many studies show a significant alteration in patients diagnosed with mental disorders: In depression, a bias toward negative emotion has been described (41–46) and hypothesized to be a predictor for relapse when distinctive (47, 48). Altered emotion recognition was also found in patients with high levels of social anxiety (49) and in children and adults with posttraumatic stress disorder (50, 51), whereas differences were no longer visible in patients with somatoform disorders when corrected for alexithymia and anxiety (52, 53).

Impulsivity is an important part and diagnostic criterion of several mental disorders (54, 55). Correlations were described regarding single aspects of the multidimensional construct of impulsivity and response inhibition, e.g., when registered with the UPPS-*P* impulsive behavior scale (56–59). A clear negative correlation was found between the aspect of urgency and response inhibition, but not in the dimensions of lack of perseverance, lack of premeditation, and sensation seeking (56, 59–61).

As most of the studies report cross-sectional data, we were interested in a longitudinal approach to see whether there are not only differences between emotional and neutral stimuli at one timepoint but also changes in response inhibition over time, especially after an inpatient and day-hospital patient treatment at a psychosomatic ward. Therefore, our study examined a population of inpatients and day-hospital patients in a psychosomatic ward to examine possible differences in response inhibition, comparing emotional and neutral stimuli and whether there is an effect of treatment.

The following hypotheses were tested: (1) The SSRT for neutral faces is slower than for negative emotional stimuli in patients with mental disorders. This was shown for patients diagnosed with schizophrenia before (19, 23). As an altered emotion recognition was shown before in different mental disorders (41–46, 49, 50, 52), we assumed that the facilitating effect of the distinct emotion of anger is seen in other mental disorders as well. (2) Response inhibition for both neutral and angry faces is increased after treatment, resulting in a faster SSRT, as an effect of treatment supporting normalization of affected response inhibition (62, 63). (3) At the baseline measurement (T0), the SSRT for neutral and angry faces differs between the individual groups of patients with different mental disorders participating in our study, as they are differences in the extent of alteration in emotion recognition as described above.

## 2. Materials and methods

### 2.1. Study design and subjects

The study was designed as a prospective, uncontrolled cohort study at the Department of Psychosomatics Medicine and Psychotherapy, University Hospital Tübingen (Universitätsklinikum Tübingen, UKT). Participants were recruited from all patients being admitted to the department either as full- or part-time inpatient and day-hospital patients between August 2019 and March 2020. Day-hospital patients spent weekdays from 8 a.m. to 4 p.m. in the clinic but went home overnight and for the weekends. The study was approved by the ethics committee of the Medical Faculty of Eberhard-Karls University Tübingen (105/2019BO2).

Inclusion criteria were regular admission for full-time or part-time treatment at the Department of Psychosomatics Medicine and Psychotherapy at the UKT, sufficient knowledge of the German language, and an age ≥ 18 years. Exclusion criteria were acute psychotic episodes, organic brain disorders, pregnancy, current dependency on alcohol or illegal drugs, and refusal to participate.

All patients who passed the inclusion criteria were asked for participation and were included if they provided written consent. Participation was voluntary, and withdrawal at any time during the study was possible and did not have any consequences on the treatment.

Data were collected at two timepoints for all participants: T0 at the beginning of the treatment within 3 days after admission as baseline. T1 was measured at the end of the treatment (within 3 days before discharge). In case of stepping down the intensity of treatment from full-time to part-time treatment to prepare for discharge, the T1 measurement took place within 3 days before the end of the full-time treatment. For the subgroup of patients with depressive disorders, we scheduled a third timepoint of assessment (T2) 1 year after discharge (within a range of ±14 days). Participants who completed all three measurements were given a gift coupon to a local bookshop as a reward (20 €, without being informed beforehand).

The initial aim of the study was to include *n* = 100 patients in the study within a timeframe of 1 year (August 2019 until August 2020). Due to the COVID-19 pandemic and resulting safety regulations recruitment had to be terminated prematurely, so only 66 patients could complete T1 measurement. Because of the premature end of the study due to COVID-19 restrictions, we conducted an interim analysis. This showed that only the group of patients with depressive disorders was large enough to conduct further statistical analyses. Therefore, we only measured the patients with depressive disorders (F32.1/2/F33.1/2) (*n* = 41) as the main diagnosis again at timepoint T2.

A total of 18 datasets of originally 119 participants had to be excluded due to violation of one or more criteria that have shown to affect reliable SSRT estimation (64). For the detailed criteria see data modeling (3.4.). After this adjustment, data from 101 participants (77 women) were included at T0, data from 57 participants (45 women) at T1, and data from 22 participants (18 women) at T2.

### 2.2. Patients and treatment

#### 2.2.1. Patients

Psychosomatic clinics in Germany treat patients with different disorders, such as somatoform disorders (ICD-10 F45.1/3/40/41 and F51.0), depressive disorders (ICD-10 F32.1/2, F33.1/2), trauma-related (ICD-10 F43.X), and anxiety disorders (ICD-10 F40.0 F41.1/2/8) as well as eating disorders (ICD-10 F50.X). The necessity of an inpatient or day-hospital patient treatment is decided at the psychosomatic outpatient clinic, where the prospect patients have an appointment ahead of potential admission. There, the patients are assessed by a clinical evaluator. Criteria for full- or part-time inpatient treatment are primary diagnosis, severity of symptoms, comorbidities and somatic consequences of the diagnosed mental disorder, dysfunctional support system or surroundings, problems with preserving daily routines, and failure of outpatient therapy.

#### 2.2.2. Treatment

All patients received the standard treatment of our department, which includes individual and group psychotherapy, music or art therapy, relaxation therapy, movement therapy, weekly ward rounds with senior physicians, and frequent therapeutic contacts with nurses, physicians, and psychologists. Existent psychopharmaceutic medication was continued, and/or psychopharmaceutic medication was started if indicated. Additionally, individual therapy elements such as nutritional counseling, biofeedback therapy, social skills training, occupational therapy, dance or gardening therapy, and family counseling were offered if indicated. Somatic diagnostic with appropriate methods was undertaken if necessary. The mean duration of treatment was 48.61 (±16.09) days.

### 2.3. Tasks

#### 2.3.1. Emotional stop-signal task

**Figure 1:**
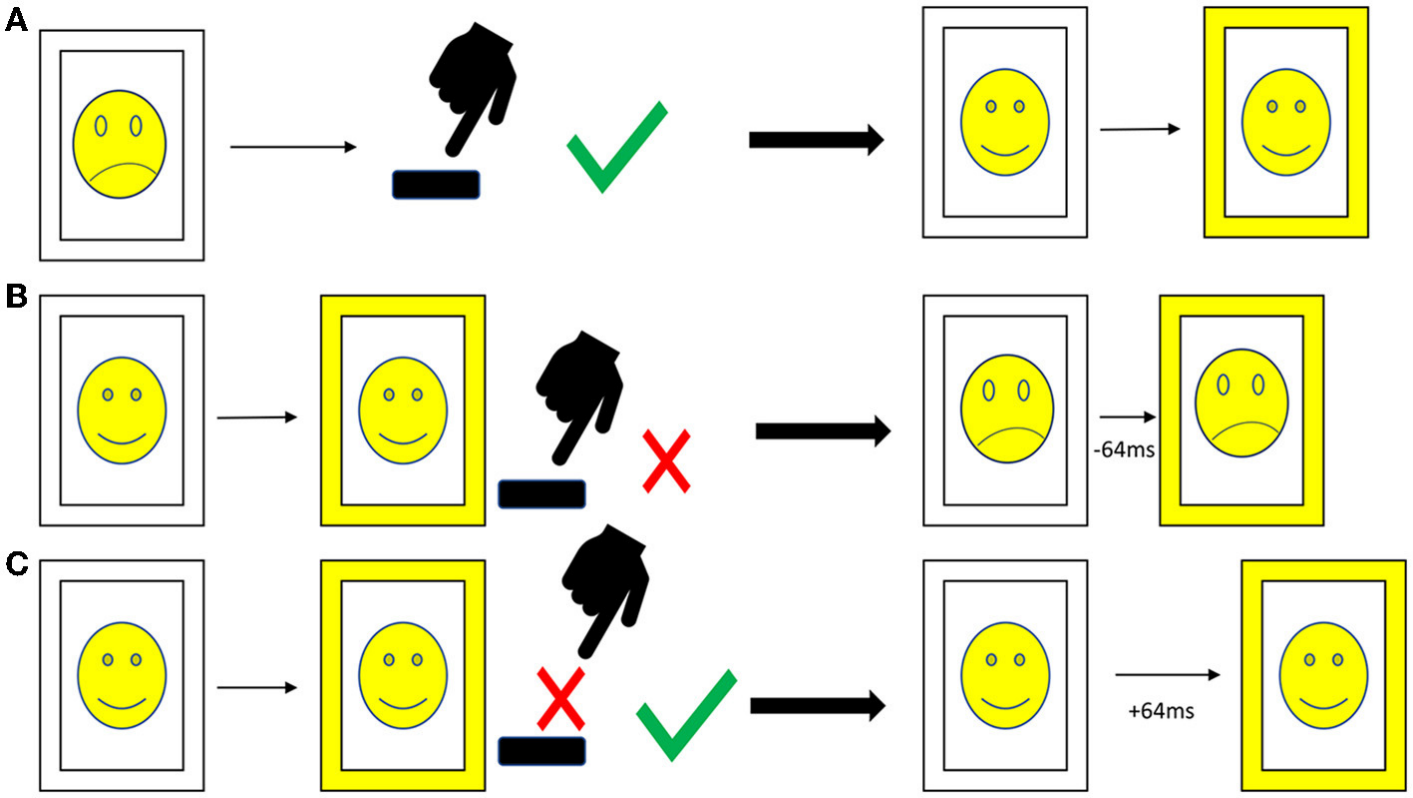
Procedure of the emotional stop-signal task. **(A)** Go trial: after the appearance of the picture with a white frame the participant presses, the space bar as fast as possible. The stop-signal delay is not affected. **(B)** Stop trial, answered incorrectly: the participant presses the space bar although there is a stop-signal (frame turns yellow); thus, in the next stop trial, the stop-signal delay is reduced by 64 ms. **(C)** Stop trial, answered correctly: the participant resists pressing the space bar correctly when the stop-signal appears; thus, the stop-signal delay is increased by 64 ms. Due to copyright reasons, the pictures in the figure are displayed as cartoons, and the pictures in the emotional stop-signal task were real photographs.

At every timepoint, patients completed an emotional stop-signal task (ESST) as described before (19). One hundred and twenty different pictures of angry and neutral faces taken from the face repository of the Brain Behavior Laboratory of the University of Pennsylvania (65) were presented in a randomized order three to four times and balanced for the sex of the depicted faces, resulting in a total of 400 trials (200 trials with angry and 200 trials with neutral faces). The content (sex and emotion of the face) was irrelevant to the task, which was to press the space bar as fast as possible when the picture appeared (go-signal), but not if there appeared a yellow frame around the picture (stop-signal). In the beginning, every picture had a white frame (see Figure 1).

To obtain a balanced ratio between correctly and incorrectly answered stop-signals, we implemented a staircase procedure for the stop-signal delay (SSD, see Figure 1). In the beginning, the SSD was set at 200 ms; with every correctly solved stop-signal, the delay increased by 64 ms; with every incorrectly answered stop-signal, the delay decreased by 64 ms. This procedure is well-established to adjust the difficulty of the stop-signal task (19, 66–69). The rate of go-signal/stop-signal was 3:1 in order to facilitate pressing the button. The patients were requested to react as fast as possible but also as accurate as possible. The pictures were visible for a maximum of 1,000 ms, and if the patient did not press within this time, the picture was registered as missing. We included two pauses of 1 min each for the patients to relax. The patients completed the task on a laptop using the Presentation^®^ software from NeuroBehavioral Systems (Berkeley, CA, USA). They received written instructions on the laptop as well as standardized verbal instructions and the possibility to ask in case of questions.

#### 2.3.2. UPPS-P impulsive behavior scale

All patients completed the German version of the UPPS impulsive behavior scale (58, 70) at all two/three measurement points to assess impulsivity. Cronbach's alpha for the present sample was 0.868 for urgency, 0.725 for lack of premeditation, 0.799 for lack of perseverance, and 0.877 for sensation seeking indicating acceptable (>0.7) or good (>0.8) internal consistency.

#### 2.3.3. PHQ-D

At all two/three measurement points patients completed a German version of the PHQ-D (71) including the subscales PHQ-9 to assess depressiveness, PHQ-15 to assess somatization, and GAD-7 to assess anxiety. Cronbach's alpha for the present sample was 0.909 for PHQ-9, 0.861 for PHQ-15, and 0.943 for GAD-7, indicating good (>0.8) or excellent (>0.09) internal consistency.

### 2.4. Data modeling of the ESST

Response times were analyzed according to the independent race model (10, 12–15). SSRT was calculated using the SSRT_med_ method as described before (10, 14). Briefly, we calculated the go-signal reaction time (RT_med_) per trial minus the median of the inhibition function, which can be calculated using linear regression to find the SSD where the response rate was 0.5. For a detailed description of the calculation, please see (10). Summing up, better response inhibition is indicated by a faster SSRT and a longer SSD (38, 40, 69). To exclude outliers due to poor performance/non-performance, we implemented criteria as described before (64, 72) to ensure sufficient data quality. We used the lenient outlier criteria, which are (1) percent inhibition on stop-trials between 25 and 75%, (2) percent go responses >60%, and (3) SSRT not negative and >50 ms (64). According to these criteria, we had to exclude 18 datasets of patients who failed in one or more conditions.

### 2.5. Statistical analysis

IBM SPSS Statistics version 28.0.0.0 was used for statistical analysis. Before further statistical analyses, we explored the missing data. We performed Little's MCAR test to exclude the possibility that the missing data are not random. For both the whole group (*p* = 0.968) and each of the subgroups with missing data [depressive disorders (*p* = 0.643), anxiety disorders (*p* = 0.997), somatoform disorders (*p* = 1.000), eating disorders (*p* = 1.000)], we showed that the missing data are at random and it is legit to impute missing data. We used monotone multiple imputations with sets of 500 iterations for each missing value. Statistical significance was set at *p* < 0.05.

Demographic data were analyzed using descriptive statistics. For an intention-to-treat (ITT) analysis, we performed data imputation for the missing T1 data from all 101 participants who completed the T0 measurement. A repeated-measures ANOVA was used to calculate the effect of emotion and time on SSRT and SSD. A Greenhouse–Geisser adjustment was applied when necessary. Additionally, we calculated a linear mixed model to examine the change of SSR for AF and NF over time as a main effect with time as a fixed effect. A random intercept model with variance components was used, and no additional random effects were applied. No other correlations or interactions were modeled. The PHQ-D-subscales were added in the per-protocol (PP) analysis as covariates. The PP analysis included all 57 participants who completed T0 and T1 measurements. Accuracy was calculated with paired *t*-tests. As the individual diagnosis groups were quite small, we only conducted PP analyses for the patients with depressive disorders with paired *t*-tests and Mann–Whitney *U*-tests in case data were not normally distributed. For all other diagnosis groups, we only reported descriptive data as the individual groups were too small. Additionally, we calculated correlations between the parameters of the ESST, the UPPS-*P* impulsive behavior scale, and the PHQ-D using Spearman's rho correlations. Additionally, we calculated in the PP analysis a linear multiple regression to examine the influence of psychopharmaceutic medication on the results.

An additional analysis was calculated for patients with depressive disorders, as these were the only diagnosis group with a third measurement point. As we had only missing data in hierarchical follow-up measurements, we were able to calculate a linear mixed model for the ITT analysis of this subgroup. We examined the change in SSRT for NF and AF over time as the main effect. The setup was the same as for the whole group. For the PP analysis, we used a repeated-measures ANOVA as we had three timepoints. A PP analysis was carried out for the results of the UPPS and the PHQ-D. Single missing data were imputed using similar response pattern imputation. To compare the results over time, we used paired *t*-tests.

## 3. Results

### 3.1. Sample characteristic

A characterization of the study population is given in Table 1.

**Table 1:** Sample characteristics.

**Parameter**	**Intention-to-treat total (%) or mean (±SD)**	**Per-protocol total (%) or mean (±SD)**
Inclusion in study	101 (100%)	
Participation in T1	57 (56.44%)	57 (100%)
Dropout	44 (43.56%)	
**Sex**		
- Male	24 (23.8%)	12 (21.1%)
- Female	77 (76.2%)	45 (78.9%)
Age (years)	41.43 (±13.13)	41.33 (±12.32)
Time between measurements (days)	47.18 (±14.51)	47.18 (±14.51)
**Primary setting**		
- Full-time inpatient	72 (71.3%)	40 (70.2%)
- Day-hospital inpatient	29 (28.7%)	17 (29.9%)
**Diagnostic category**		
- Depressive disorders	41 (40.6%)	28 (49.1%)
- Somatoform disorders	31 (30.7%)	13 (22.8%)
- Eating disorders	8 (7.9%)	3 (5.3%)
- Anxiety and trauma	21 (20.8%)	13 (22.8%)
**Subgroup depressive disorders**		
- Inclusion in study	41 (100%)	
- Participation in T1	28 (68.29%)	28 (100%)
- Participation in T2	22 (53.66%)	22 (78.57%)
- Dropout	23 (56.10%)	10 (35.71%)
- Time between T1 and T1 (days)	46.96 (±16.60)	45.36 (±13.38)
- Time between T1 and T2 (days)	371.56 (±8.77)	371.56 (±8.77)

### 3.2. Results for the whole group

**Figure 2:**
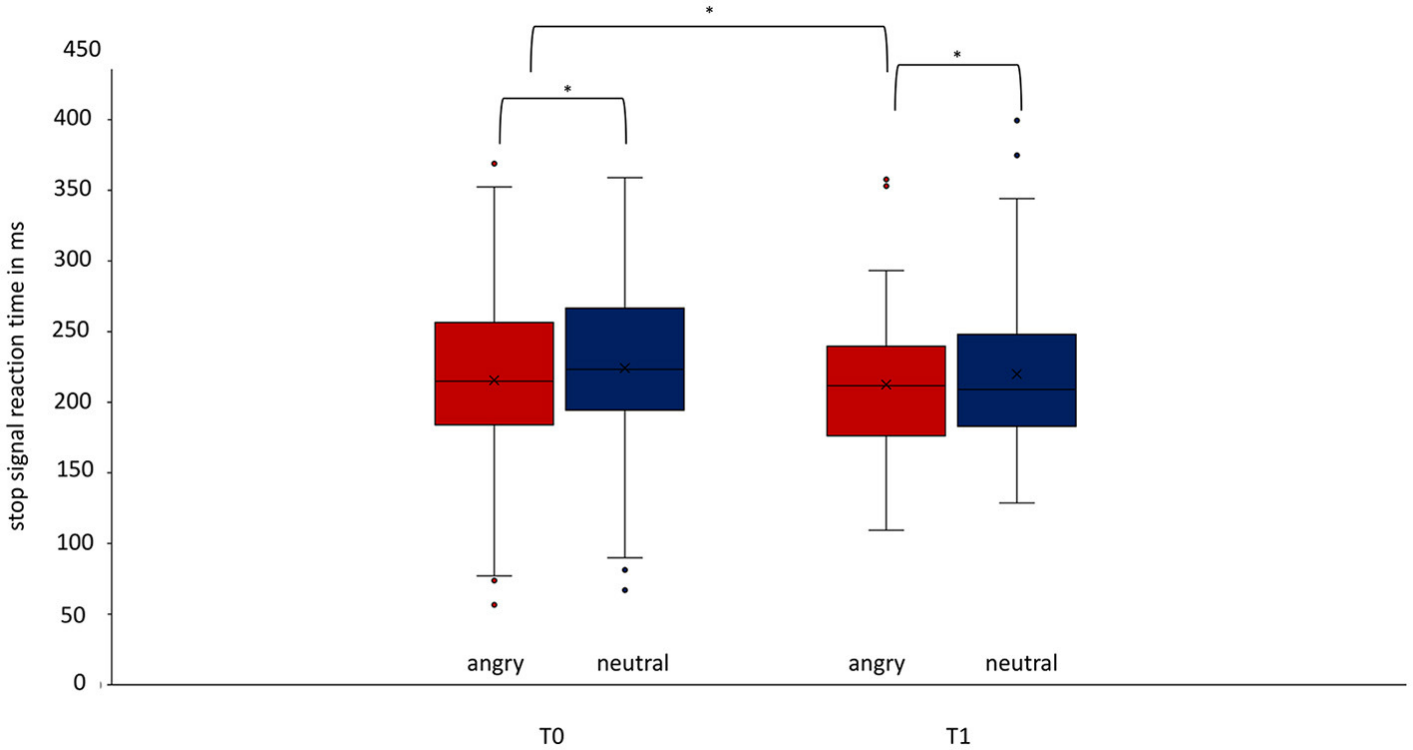
Difference of the stop-signal reaction time for angry and neutral faces at timepoints T0 and T1 regarding the whole group. At both timepoints T0 (*p* < 0.001, Cohen's *d* = −0.278) and T1 (*p* < 0.001, Cohen's *d* = −0.320), there is a significant difference in stop-signal reaction time for angry and neutral stimuli. The SSRT improved significantly from T0 to T1 for angry faces treatment (*p* = 0.011). Data are expressed as mean ± SD. T0, baseline measurement within 3 days after admission; T1, discharge measurement within 3 days prior to discharge. ^*^p < 0.05.

In the ITT analysis, the rmANOVA, corrected with a Greenhouse–Geisser adjustment because of violation of sphericity, showed significant differences in the SSRT [*F*_(1.453, 815.089)_ = 17.187, *p* = < 0.001, part. η^2^ = 0.030]. Bonferroni-corrected *post-hoc* analysis showed significantly faster SSRT for angry than neutral faces at T0 (*p* < 0.001) and T1 (*p* < 0.001) (Figure 2) and a significantly faster SSRT at timepoint T1 than at timepoint T0 for angry faces (*p* = 0.0110) but not for neutral faces (*p* = 0.250). For SSD, the rmANOVA again showed a main effect of emotion, a main effect of time, and a significant interaction of emotion and time [*F*_(1.029, 577.015)_ = 5.734, *p* = 0.016, η^2^ = 0.010]. Bonferroni-corrected *post-hoc* analysis showed significant differences in SSD between angry neutral faces at T0 (*p* < 0.001) and T1 (*p* < 0.001). Furthermore, the ITT analysis showed significant differences between the accuracy of stop-trials between angry and neutral faces at T0 [*T*_(100)_ = −2.937, *p* = 0.003], with a trend at T1 [*T*_(100)_ = −1.959, *p* = 0.050]. The linear mixed model did not show these effects. There was no significant difference between timepoints T0 and T1, neither for neutral (*p* = 0.469) nor for angry faces (*p* = 0.126). In the PP analysis, the repeated-measures ANOVA showed no differences in the SSRT regarding emotion or time [*F*_(1.485, 83.145)_ = 1.544, *p* = 0.222]. There was no significant difference for all other parameters investigated: accuracy of go-trials in total, accuracy of go-trials in case of neutral stimuli, accuracy of go-trials in case of angry stimuli, median reaction time in go-tasks (RTmed) in case of neutral stimuli or RTmed in case of angry stimuli (Table 2; Supplementary Table 1).

**Table 2:** Performance of the whole group in the emotional stop-signal task (*n* = 101).

**Parameter**	**T0**	***p*_e_**	**T1**	***p*_e_**	***p*_t_**
**Accuracy of stop-trials (%)**					
Total	52.09%		51.67%		0.201
Anger	50.48%		49.55%		0.415
Neutral	53.7%	**0.003**	53.78%	**0.050**	0.947
**SSRT (ms, mean** ±**SD)**					
Anger	215.958 (±55.09)		207.473 (±68.60)		**0.011**
Neutral	224.496 (±59.66)	**< 0.001**	218.615 (±64.16)	**< 0.001**	0.250
**SSD (ms, mean** ±**SD)**					
Anger	419.75 (±150.45)		429.88 (±193.50)		0.451
Neutral	411.39 (±145.96)	**< 0.001**	422.93 (±187.24)	**< 0.001**	0.190

Intention-to-treat analysis of the data; SSD, stop-signal delay; SSRT, stop-signal reaction time; T0, baseline measurement; T1, discharge measurement; *p*_e_ *p*, emotion compared; *p*_t_ *p*, time compared. Significant *p*-values are displayed in bold.

### 3.3. Results for different diagnosis groups

#### 3.3.1. Differences between emotional and neutral faces at one measurement point

The results for patients with depressive disorders are reported at point 3.3.2. All other groups were quite small, so only descriptive data are reported (Supplementary Tables 2, 3).

#### 3.3.2. Differences between the single measurement points

**Figure 3:**
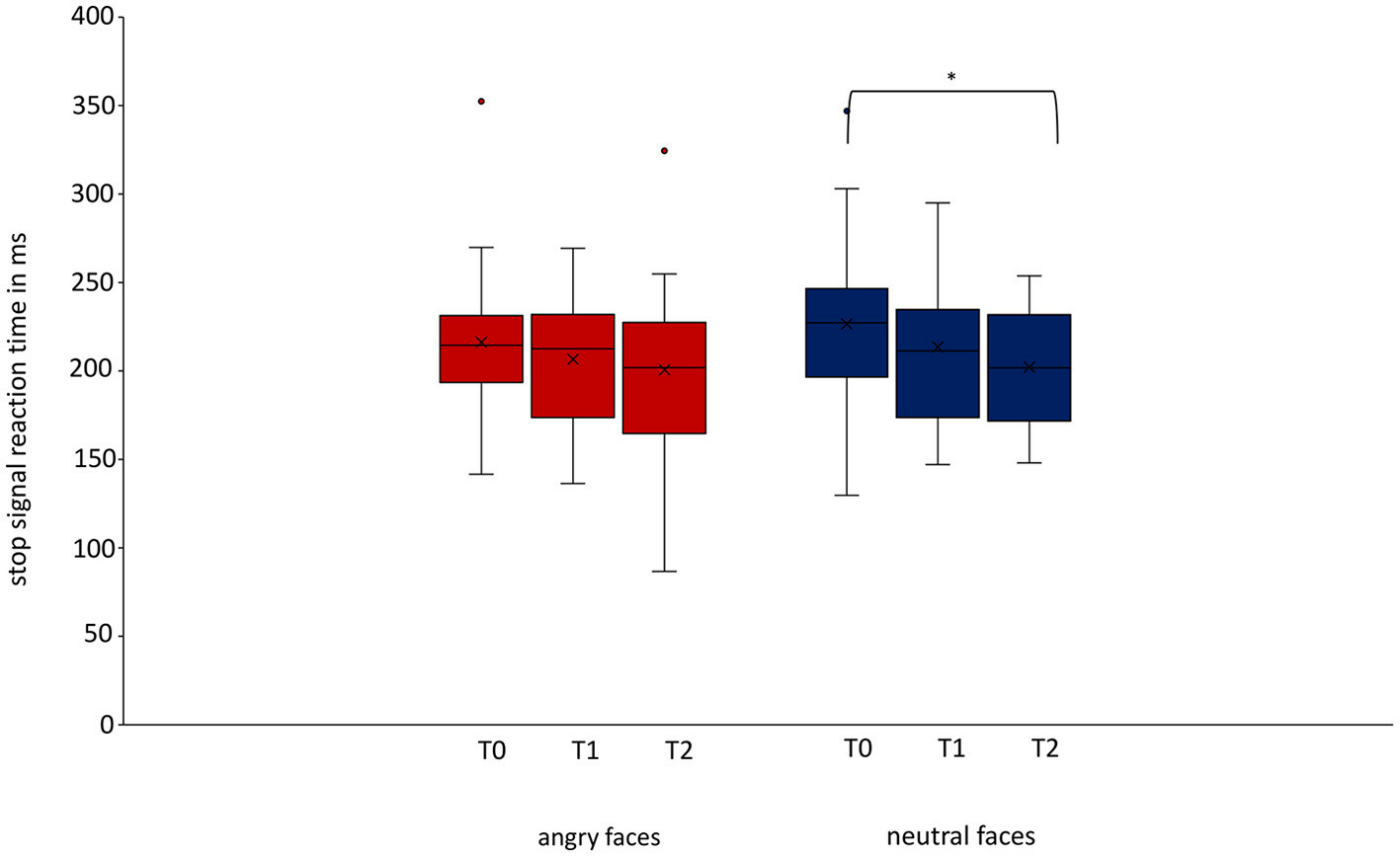
Change of stop-signal reaction time over time for angry and neutral faces in patients with depressive disorders. For patients with depressive disorders, we could show a decrease in stop-signal reaction time for neutral stimuli between baseline and follow-up measurement was significant (T0/T2 *p* = 0.038, Cohen's *d* = 0.631). Data are expressed as mean ± SD. T0, baseline measurement within 3 days after admission; T1, discharge measurement within 3 days prior to discharge; T2, follow-up measurement 1 year (±7 days) after discharge. ^*^p < 0.05.

The linear mixed model for the ITT analysis in the group of patients with depressive disorders showed a significant difference in SSRT for neutral faces between T0 and T2 (*p* = 0.038) but not between T0 and T1 (*p* = 0.386) or between T1 and T2 (*p* = 0.283; Figure 3; Table 3). There was no significant difference for SSRT for angry faces at any timepoint (T0/T1: *p* = 0.651; T0/T2 *p* = 0.318). Even though there was no significant difference between T0 and T1, a continuous decrease in SSRT for neutral stimuli from T0 over timepoint T1 to timepoint T2 was observed (Table 4). The rmANOVA in the PP analysis showed a significant difference between the timepoints for neutral faces [*F*_(2, 42)_ = 3.824, *p* = 0.030] but not for angry faces [*F*_(2, 42)_ = 0.919, *p* = 0.407]. Bonferroni-corrected *post-hoc* analysis for neutral faces showed a significant difference (*p* = 0.020) between T0 and T2. For the other diagnosis groups, somatoform disorders, eating disorders, and anxiety and trauma, we only reported descriptive data, because of the small number of participants (Supplementary Table 2).

**Table 3:** Results for patients with depressive disorders (T0: *n* = 41, T1: *n* = 28, T2: *n* = 22).

**Parameter**	**T0**	**T1**	**T2**	***p*_e_ T0**	***p*_e_ T1**	***p*_e_ T2**	***p*_t_ T0/T1**	***p*_t_ T0/T2**	***p*_t_ T1/T2**
**Accuracy of stop-trials (%)**									
Total	52.04%	51.57%	51.81%				0.108	0.480	0.199
Anger	50.86%	50.5%	48.27%				0.810	0.131	0.404
Neutral	53.21%	52.64%	55.36%	0.066	0.394	**0.003**	0.711	0.243	0.255
**SSRT (ms, mean** ±**SD)**									
Anger	207.08 (±58.98)	207.55 (±47.39)	200.60 (±49.11)				0.328	0.236	0.541
Neutral	214.90 (±57.97)	212.03 (±50.71)	202.07 (±33.58)	0.096	0.371	0.835	0.153	**0.003**	0.110
**SSD (ms, mean** ±**SD)**									
Anger	436.96 (±169.09)	419.58 (±162.18)	420.19 (±130.36)				0.465	0.305	0.755
Neutral	427.93 (±164.32)	414.29 (±159.15)	417.43 (±128.58)	**0.003**	**0.007**	0.057	0.309	0.209	0.648

Per-protocol analysis of the data; SSRT, stop-signal reaction time; SSD, stop-signal delay; T0, baseline measurement; T1, discharge measurement; T2, follow-up measurement 1 year after discharge; *p*_e_ *p*, emotion compared; *p*_t_ *p*, time compared. Significant *p*-values are displayed in bold.

**Table 4:** Results over time for patients with depressive disorders.

**Timepoint**	**SSRT (ms)**	***p* when compared with T1**	***p* when compared with T2**
**Anger**			
T0	207.075	0.651	0.318
T1	202.462		0.461
T2	193.606	0.461	
**Neutral**			
T0	214.896	0.386	**0.038**
T1	205.891		0.283
T2	195.881	0.283	

Data were analyzed with a linear mixed model; SSRT, stop-signal reaction time; T0, baseline measurement; T1, discharge measurement; T2, follow-up measurement 1 year after discharge. Significant *p*-values are displayed in bold.

### 3.4. UPPS-P impulsive behavior scale results

The PP analysis showed a relatively stable self-report of urgency parameters for the whole group as there were no significant changes over treatment. In the group of patients with depressive disorders, the parameter lack of premeditation increased significantly from 21.65 (SD 5.244) points at T0 to 23.25 (SD 4.387) points at T2 [*t*_(19)_ = −1.6, *p* = 0.044]. There were no other significant changes over time (Table 5).

**Table 5:** UPPS impulsive behavior scale.

**Time point**	**Urgency**	***p* (T0/T1)**	***p* (T0/T2)**	***p* (T1/T2)**	**Lack of premeditation**	***p* (T0/T1)**	***p* (T0/T2)**	***p* (T1/T2)**	**Lack of perseverance**	***P* (T0/T1**	***p* (T0/T2)**	***p* (T1/T2)**	**Sensation seeking**	***p* (T0/T1**	***p* (T0/T2)**	***p* (T1/T2)**
**Whole group (*****n*** = **54)**																
T0	28.31 (±7.03)				21.98 (±5.10)				20.78 (±5.65)				24.46 (±8.11)			
T1	27.98 (±7.23)	0.577			22.59 (±4.82)	0.278			19.91 (±4.73)	0.086			24.72 (±7.66)	0.599		
**Depressive disorders (*****n*** = **20)**																
T0	29.65 (±7.34)				21.65 (±5.24)				22.6 (±5.21)				24.05 (±8.49)			
T1	30.40 (±6.96)	0.406			22.50 (±3.98)	0.439			20.95 (±3.39)	0.116			24.4 (±7.42)	0.725		
T2	29.35 (±6.24)		0.777	0.29	23.25 (±4.39)		**0.044**	0.412	20.9 (±3.74)		0.058	0.952	23.75 (±7.8)		0.787	0.402
**Somatoform disorders (*****n*** = **13)**																
T0	26.54 (±5.74)				20.31 (±5.33)				19.92 (±6.26)				24.85 (±8.23)			
T1	25.31 (±6.26)				22.31 (±5.69)				18.69 (±5.39)				25.23 (±8.69)			
**Eating disorders (*****n*** = **3)**																
T0	30.00 (±10.58)				22.00 (±5.29)				17.67 (±6.03)				26.33 (±8.62)			
T1	24.67 (±7.64)				23.33 (±5.69)				17.67 (±6.66)				28.33 (±9.50)			
**Anxiety and trauma (*****n*** = **13)**																
T0	26.61 (±8.04)				22.54 (±3.92)				18.85 (±4.00)				24.77 (±9.36)			
T1	27.08 (±7.90)				23.23 (±6.07)				19.15 (±4.81)				24.62 (±7.94)			

Data were analyzed as per the protocol. Significant *p*-values are displayed in bold.

### 3.5. PHQ-D results

The PP analysis showed a significant decrease in symptom severity for the whole group over treatment in all three scales: PHQ-9 (*p* = 0.001), PHQ-15 (*p* = 0.036), and GAD-7 (*p* = 0.001; Table 6). In the group of patients with depressive disorders, PHQ-9 (*p* = 0.006) and GAD-7 (*p* = 0.0.24) decreased significantly between T0 and T1 and PHQ-15 increased significantly from T1 to T2 (*p* = 0.042; Table 6).

**Table 6:** Patient health questionnaire, German version (PHQ-D).

**Time point**	**PHQ-9**	***p* (T0/T1)**	***p* (T0/T2)**	***p* (T1/T2)**	**PHQ-15**	***p* (T0/T1)**	***p* (T0/T2)**	***p* (T1/T2)**	**GAD-7**	***p* (T0/T1**	***p* (T0/T2)**	***p* (T1/T2)**
**Whole group (*****n*** = **57)**												
T0	12.65 (±6.37)				8.44 (±4.53)				9.21 (±5.62)			
T1	9.68 (±5.69)	**0.001**			7.56 (±4.18)	**0.036**			6.75 (±5.44)	**0.001**		
**Depressive disorders (*****n*** = **22)**												
T0	14.45 (±6.50)				8.91 (±5.21)				11.14 (±5.42)			
T1	9.91 (±5.66)	**0.006**			7.36 (±4.09)	0.073			8.05 (±5.96)	**0.024**		
T2	10.68 (±8.18)		0.108	0.510	11.60 (±9.51)		0.265	**0.042**	8.64 (±6.83)		0.207	0.629
**Somatoform disorders (*****n*** = **13)**												
T0	11.46 (±5.80)				8.77 (±4.13)				6.54 (±5.78)			
T1	9.15 (±4.13)	**0.029**			7.77 (±4.21)	0.273			4.69 (±5.01)	**0.038**		
**Eating disorders (*****n*** = **3)**												
T0	11.67 (±9.07)				6.67 (±5.03)				9.67 (±7.09)			
T1	10.33 (±6.03)	not performed			5.33 (±4.04)	Not performed			4.67 (±4.16)	Not performed		
**Anxiety and trauma (*****n*** = **13)**												
T0	10.23 (±6.64)				8.23 (±4.60)				9.08 (±6.06)			
T1	9.85 (±6.65)	0.675			8.31 (±4.84)	0.888			7.85 (±5.74)	0.377		

Data were analyzed per protocol. Significant *p*-values are displayed in bold.

### 3.6. Correlations between ESST parameters, UPPS-P, and PHQ-D

The PP analysis showed strong correlations between the single ESST parameters and the single PHQ-subscales. No significant correlations were found between the ESST parameters and the UPPS-P scale or the ESST parameters and the PHQ-D scales. Weak-to-moderate correlations were found between the UPPS-P parameter urgency and the single PHQ-D subscales as well as between GAD-7 and the UPPS-P parameter lack of perseverance (Table 7).

**Table 7:** Correlations between ESST, UPPS, and PHQ-D.

**Parameter**	**SSRT angry faces**	**SSRT neutral faces**	**SSD angry faces**	**SSD neutral faces**	**UPPS urgency**	**UPPS lack of premediation**	**UPPS lack of perseverance**	**UPPS sensation seeking**	**PHQ-9**	**PHQ-15**	**GAD-7**
**T0**											
SSRT angry faces		**0.838**, ***p*** **=** **0.001**	**0.620**, ***p*** **=** **0.001**	**0.612**, ***p*** **=** **0.001**	0.034, *p* = 0.902	0.071, *p* = 0.600	0.016, *p* = 0.903	0.136, *p* = 0.312	0.010, *p* = 0.931	0.021, *p* = 0.859	0.026, *p* = 0.828
SSRT neutral faces	**0.838**, ***p*** **=** **0.001**		**0.525**, ***p*** **=** **0.001**	**0.565**, ***p*** **=** **0.001**	0.802, *p* = 0.527	0.122, *p* = 0.365	0.007, *p* = 0.962	0.241, *p* = 0.241	0.062, *p* = 0.598	0.011, *p* = 0.928	0.058, *p* = 0.621
SSD angry faces	**0.620**, ***p*** **=** **0.001**	**0.525**, ***p*** **=** **0.001**		**0.989**, ***p*** **=** **0.001**	0.174, *p* = 0.195	0.157, *p* = 0.244	0.041, *p* = 0.760	0.013, *p* = 0.923	0.118, *p* = 0.319	0.190, *p* = 0.104	0.090, *p* = 0.447
SSD neutral faces	**0.612**, ***p*** **=** **0.001**	**0.565**, ***p*** **=** **0.001**	**0.989**, ***p*** **=** **0.001**		0.164, *p* = 0.224	0.158, *p* = 0.241	0.044, *p* = 0.745	0.010, *p* = 0.940	0.108, *p* = 0.359	0.198, *p* = 0.090	0.095, *p* = 0.420
UPPS urgency	0.034, *p* = 0.828	0.085, *p* = 0.527	0.174, *p* = 0.195	0.164, *p* = 0.224		0.191, *p* = 0.154	**0.372**, ***p*** **=** **0.004**	0.017, *p* = 0.901	**0.407**, ***p*** **=** **0.002**	**0.324**, ***p*** **=** **0.014**	**0.295**, ***p*** **=** **0.026**
UPPS lack of, premediation	0.071, *p* = 0.600	0.122, *p* = 0.365	0.157, *p* = 0.244	0.158, *p* = 0.241	0.191, *p* = 0.154		**0.435**, ***p*** **=** **0.001**	0.180, *p* = 0.180	0.168, *p* = 0.211	0.101, *p* = 0.456	0.179, *p* = 0.182
UPPS lack of, perseverance	0.016, *p* = 0.903	0.007, *p* = 0.962	0.041, *p* = 0.760	0.044, *p* = 0.745	**0.372**, ***p*** **=** **0.004**	**0.435**, ***p*** **=** **0.001**		0.104, *p* = 0.443	**0.330**, ***p*** **=** **0.012**	0.190, *p* = 0.158	**0.350**, ***p*** **=** **0.008**
UPPS sensation seeking	0.136, *p* = 0.312	0.241, *p* = 0.071	0.013, *p* = 0.923	0.010, *p* = 0.940	0.017, *p* = 0.901	0.180, *p* = 0.180	0.104, *p* = 0.443		0.141, *p* = 0.296	**0.308**, ***p*** **=** **0.020**	**0.285**, ***p*** **=** **0.032**
PHQ-9	0.010, *p* = 0.931	0.062, *p* = 0.598	0.118, *p* = 0.319	0.108, *p* = 0.359	**0.407**, ***p*** **=** **0.002**	0.168, *p* = 0.211	**0.330**, ***p*** **=** **0.012**	0.141, *p* = 0.296		**0.557**, ***p*** **=** **0.001**	**0.806**, ***p*** **=** **0.001**
PHQ-15	0.021, *p* = 0.859	0.011, *p* = 0.928	0.190, *p* = 0.104	0.198, *p* = 0.090	**0.324**, ***p*** **=** **0.014**	0.101, *p* = 0.456	0.190, *p* = 0.158	**0.308**, ***p*** **=** **0.020**	**0.557**, ***p*** **=** **0.001**		**0.606**, ***p*** **=** **0.001**
GAD-7	0.026, *p* = 0.828	0.058, *p* = 0.621	0.090, *p* = 0.447	0.095, *p* = 0.420	**0.295**, ***p*** **=** **0.026**	0.179, *p* = 0.182	**0.350**, ***p*** **=** **0.008**	**0.285**, ***p*** **=** **0.032**	**0.806**, ***p*** **=** **0.001**	**0.606**, ***p*** **=** **0.001**	
**T1**											
SSRT angry faces		**0.849**, ***p*** **=** **0.001**	**0.627**, ***p*** **=** **0.001**	**0.629**, ***p*** **=** **0.001**	0.084, *p* = 0.548	0.181, *p* = 0.191	0.169, *p* = 0.223	0.170, *p* = 0.219	0.114, *p* = 0.400	0.221, *p* = 0.098	0.130, *p* = 0.336
SSRT neutral faces	**0.849**, ***p*** **=** **0.001**		**0.595**, ***p*** **=** **0.001**	**0.613**, ***p*** **=** **0.001**	0.137, *p* = 0.323	0.222, *p* = 0.106	0.179, *p* = 0.195	0.119, *p* = 0.391	0.036, *p* = 0.789	0.121, *p* = 0.370	0.019, *p* = 0.0.886
SSD angry faces	**0.627**, ***p*** **=** **0.001**	**0.595**, ***p*** **=** **0.001**		**0.996**, ***p*** **=** **0.001**	0.053, *p* = 0.704	0.098, *p* = 0.481	0.015, *p* = 0.0.917	0.025, *p* = 0.856	0.120, *p* = 0.375	0.207, *p* = 0.123	0.031, *p* = 0.818
SSD neutral faces	**0.629**, ***p*** **=** **0.001**	**0.613**, ***p*** **=** **0.001**	**0.996**, ***p*** **=** **0.001**		0.054, *p* = 0.698	0.103, *p* = 0.460	0.003, *p* = 0.981	0.019, *p* = 0.893	0.136, *p* = 0.316	0.220, *p* = 0.101	0.040, *p* = 0.769
UPPS urgency	0.084, *p* = 0.548	0.137, *p* = 0.323	0.053, *p* = 0.704	054, *p* = 0.698		0.077, *p* = 0.582	0.**362**, ***p*** **=** **0.007**	0.008, *p* = 0.956	**0.428**, ***p*** **=** **0.001**	**0.396**, ***p*** **=** **0.003**	**0.443**, ***p*** **=** **0.001**
UPPS lack of, premediation	0.181, *p* = 0.0.191	0.222, *p* = 0.106	0.098, *p* = 0.481	0.103, *p* = 0.460	0.077, *p* = 0.582		**0.429**, ***p*** **=** **0.001**	0.123, *p* = 0.375	0.169, *p* = 0.223	0.105, *p* = 0.448	0.070, *p* = 0.615
UPPS lack of, perseverance	0.169, *p* = 0.223	0.179, *p* = 0.195	0.015, *p* = 0.917	0.003, *p* = 0.981	**0.362**, ***p*** **=** **0.007**	**0.429**, ***p*** **=** **0.001**		0.023, *p* = 0.867	0.259, *p* = 0.059	0.158, *p* = 0.255	**0.316**, ***p*** **=** **0.020**
UPPS sensation seeking	0.170, *p* = 0.219	0.119, *p* = 0.391	0.025, *p* = 0.856	0.019, *p* = 0.893	0.008, *p* = 0.956	0.123, *p* = 0.375	0.023, *p* = 0.867		0.097, *p* = 0.483	0.134, *p* = 0.335	0.075, *p* = 0.589
PHQ-9	0.114, *p* = 0.400	0.036, *p* = 0.789	0.120, *p* = 0.375	0.135, *p* = 0.316	**0.428**, ***p*** **=** **0.001**	0.169, *p* = 0.223	0.259, *p* = 0.059	0.097, *p* = 0.483		**0.550**, ***p*** **=** **0.001**	**0.810**, ***p*** **=** **0.001**
PHQ-15	0.221, *p* = 0.098	0.121, *p* = 0.370	0.207, *p* = 0.123	0.220, *p* = 0.101	**0.396**, ***p*** **=** **0.003**	0.105, *p* = 0.448	0.158, *p* = 0.255	0.134, *p* = 0.335	**0.550**, ***p*** **=** **0.001**		**0.568**, ***p*** **=** **0.001**
GAD-7	0.130, *p* = 0.336	0.019, *p* = 0.886	0.031, *p* = 0.818	0.040, *p* = 0.769	**0.443**, ***p*** **=** **0.001**	0.070, *p* = 0.615	**0.316**, ***p*** **=** **0.020**	0.075, *p* = 0.589	**0.810**, ***p*** **=** **0.001**	**0.568**, ***p*** **=** **0.001**	

Data were analyzed per protocol. Significant *p*-values are displayed in bold. Correlations were tested with Spearman's rho correlation.

### 3.7. Influence of psychopharmaceutic medication

We had data about psychopharmaceutic medication from 47 of the 57 patients included in the PP analysis. Twenty-four of them (42.1%) had no psychopharmaceutic medication at all, 11 (23.4%) used drugs from exactly one medication group listed in Supplementary Table 3, and 12 (25.5%) used drugs from more than one drug group. The linear regression showed no influence of medication in general on the SSRT, neither for angry faces [*F*_(1, 45)_ = 0.864, *p* = 0.358] or neutral faces [*F*_(1, 45)_ = 1.523, *p* = 0.224] at T0 nor for angry [*F*_(1, 45)_ = 0.090, *p* = 0.766] or neutral faces [*F*_(1, 45)_ = 0.098, *p* = 0.756] at T1. In an additional analysis, no differences were found regarding the single medication groups at T0 {angry: [*F*_(6, 40)_ = 0.701, *p* = 0.650], neutral: [*F*_(6, 40)_ = 1.523, *p* = 0.196]} or T1 {angry: [*F*_(6, 40)_ = 1.327, *p* = 0.268], neutral: [*F*_(6, 40)_ = 1.466, *p* = 0.215]}. See Supplementary Table 3 for detailed information on the used drugs.

## 4. Discussion

The aim of our study was to investigate a possible change of response inhibition over time after a psychosomatic inpatient and day-hospital patient treatment via an emotional stop-signal task, showing angry and neutral faces. To the best of our knowledge, this is the first study investigating the effect of psychosomatic inpatient and day-hospital patient treatment on response inhibition with an emotional stop-signal task.

We observed a difference in SSRT for neutral and angry faces with a slower SSRT for neutral faces than for emotional faces at both timepoints, before and after treatment, indicating poorer inhibitory control regarding neutral faces. This is in line with former studies that showed an altered response inhibition or emotion recognition in patients with mental disorders (12, 16–28, 38, 69). Our group argued before that the slower SSRT for neutral stimuli in schizophrenia patients might be due to the harder-to-recognize neutral faces, and thus, participants were distracted from the actual task and hesitated to press the button (19) in comparison with the easier-to-recognize and classify emotional faces (19, 23). Unfortunately, we did not collect any data about emotion recognition rates for our participants. In our study, we detected this alteration also for patients with other mental disorders. With these findings, we proved our first hypothesis of a slower SSRT for neutral than emotional stimuli in patients with psychosomatic diseases.

The second hypothesis, a better response inhibition for neutral and emotional stimuli after treatment, indicated by a faster SSRT after treatment, could not be confirmed for both angry and neutral stimuli. The repeated-measures ANOVA showed a significantly better response inhibition for angry faces at T1 compared with T0, indicated by a faster SSRT, but no significant improvement in response inhibition for neutral faces. We showed a significant decrease in symptom severity for depression, somatization, and anxiety after treatment, which is a sign of the effectiveness of the treatment and maybe a hint that the improvement in response inhibition is, at least partly, based on the effect of therapy. Furthermore, we showed a correlation between the UPPS-P subscale urgency, which has been shown to correlate to response inhibition before, and all three PHQ-D subscales before and after treatment. This finding is in line with previous studies showing an improvement in brain function after psychotherapeutic or psychopharmaceutic treatment (73). One could show an improvement in regions that are responsible for emotion recognition and response inhibition in patients with depressive disorders after cognitive behavioral therapy and/or paroxetine treatment (74). Another one showed an improvement after short-term psychodynamic therapy for patients with panic disorders (75) and another for patients with generalized anxiety disorder after mindfulness training (76). A test–retest reliability for the ESST was shown before (77), indicating that our results cannot simply be explained with learning effects.

Divergent from the results for the whole group, patients with depressive disorders displayed a faster SSRT for neutral faces at the follow-up measurement compared with the baseline measurement. The SSRT got faster at every timepoint, and only between these two timepoints and for neutral faces, the difference reached significance. An explanation may be a remission of the disorder after 1 year in a large subset of the patients, but the PHQ-9 showed a stable symptom severity between discharge and 1 year after discharge. An explanation may be a normalization of brain function in remission, as shown before (78, 79). However, this would not explain why only the improvement in response inhibition for neutral faces reached significance. Other studies showed impairment in executive functions even in remission (80–82), giving rise that only a part of executive functions is normalized in remission. This should be investigated in further studies using imaging methods. This study now provides a first hint that inpatient and day-hospital patient psychosomatic treatment leads to a better response inhibition, which is, for patients with depressive disorders, not only stable but improves further over at least a year. Further studies should investigate why there is a difference in improvement between emotional and neutral stimuli and why they do not improve equally.

Regarding our third hypothesis about a difference in SSRT between the different diagnosis groups, we did not find significant differences in SSRT or any other parameter of the ESST. The main reason could be the small sample size and high dropout rate in some of the groups because of the premature end of the study due to the COVID-19 pandemic. Therefore, further studies investigating the different diagnosis groups individually might be interesting as our sample was too small to find differences in the different groups.

In comparison with similar studies with healthy probands, we had to exclude a large group of participants (*n* = 18) due to poor results, indicating that they did not participate attentively. It was shown before that the motivation and goal of the probands can affect the results (83). Several patients reported after the test that it was challenging and frustrating for them. For some, this was a reason for dropping off the study; others may have changed their strategy or stopped participating actively. As this was not reported before for healthy participants, this may be part of their disease, as patients diagnosed with mental disorders are known for having high dropout rates due to attitudinal barriers (84–86), lower attention (87), and lower concentration being part of the ICD-10 diagnosis criteria for depressive disorders (88). As the study was designed as a prospective uncontrolled cohort study, the biggest limitation is the missing of a healthy control group, which makes it difficult to compare the result to healthy people and decide whether the described effects over time are due to learning effects or due to therapy. Therefore, this study can just hint toward an altered response inhibition when combined with emotional stimuli in patients with several mental disorders. Furthermore, bigger and more controlled studies are necessary to compare the different diagnosis groups and prove the alteration in comparison with healthy controls. However, as described above before, there are results that describe test–retest reliability (77).

In summary, in our study we showed a significant difference in SSRT for neutral and angry stimuli in a psychosomatic inpatient and day-hospital patient population, indicating a better response inhibition when a task is combined with a task-irrelevant negative emotional stimulus, as they may be easier to read and therefore less distracting. After treatment, we detected a significant improvement in response inhibition after treatment for angry faces and a faster, but not significant, SSRT for neutral faces, which might point toward a treatment effect, as symptom severity decreased significantly. However, as this study was performed as an uncontrolled prospective study we cannot say for sure that the observed changes are due to treatment, and therefore, further controlled studies are needed. For patients with depressive disorders, a significant improvement in response inhibition for neutral faces after 1 year was observed, which might be a treatment effect as well or a sign of remission.

## Data availability statement

The raw data supporting the conclusions of this article will be made available by the authors, without undue reservation.

## Ethics statement

The studies involving human participants were reviewed and approved by Medical Faculty of Eberhard-Karls University Tübingen. The patients/participants provided their written informed consent to participate in this study.

## Author contributions

SW was responsible for data collection, preparation of data for analysis, data analysis, data interpretation, and mainly drafted the paper. KS was responsible for inauguration in the ESST. BD was responsible for the conception and creation of the ESST. AS was responsible for conception and design of the study as well as data interpretation. SZ was responsible for conception and design of the study. All authors revised the manuscript and approved the final version.

## References

[1] Kalanthroff E Cohen N Henik A. Stop feeling: inhibition of emotional interference following stop-signal trials. Front Hum Neurosci. (2013) 7:78. 10.3389/fnhum.2013.00078 23503817 PMC3596782

[2] Dennis TA Chen CC McCandliss BD. Threat-related attentional biases: an analysis of three attention systems. Depress Anxiety. (2008) 25:E1–E10. 10.1002/da.20308 17565734 PMC2662699

[3] Hart SJ Green SR Casp M Belger A. Emotional priming effects during stroop task performance. Neuroimage. (2010) 49:2662–70. 10.1016/j.neuroimage.2009.10.076 19883772 PMC2818423

[4] Padmala S Bauer A Pessoa L. Negative emotion impairs conflict-driven executive control. Front Psychol. (2011) 2:192. 10.3389/fpsyg.2011.00192 21886635 PMC3154405

[5] Birk JL Dennis TA Shin LM Urry HL. Threat facilitates subsequent executive control during anxious mood. Emotion. (2011) 11:1291–304. 10.1037/a0026152 22059518

[6] Kanske P. On the influence of emotion on conflict processing. Front Integr Neurosci. (2012) 6:42. 10.3389/fnint.2012.00042 22876220 PMC3410615

[7] Kanske P Kotz SA. Emotion speeds up conflict resolution: a new role for the ventral anterior cingulate cortex? Cereb Cortex. (2011) 21:911–9. 10.1093/cercor/bhq157 20732901

[8] Kanske P Kotz SA. Emotion triggers executive attention: anterior cingulate cortex and amygdala responses to emotional words in a conflict task. Hum Brain Mapp. (2011) 32:198–208. 10.1002/hbm.21012 20715084 PMC6870409

[9] Tiego J Testa R Bellgrove MA Pantelis C Whittle S. A hierarchical model of inhibitory control. Front Psychol. (2018) 9:1339. 10.3389/fpsyg.2018.01339 30123154 PMC6085548

[10] Band GPH van der Molen MW Logan GD. Horse-race model simulations of the stop-signal procedure. Acta Psychol. (2003) 112:105–42. 10.1016/S0001-6918(02)00079-3 12521663

[11] Diamond A. Executive functions. Annu Rev Psychol. (2013) 64:135–68. 10.1146/annurev-psych-113011-143750 23020641 PMC4084861

[12] Verbruggen F Logan GD. Response inhibition in the stop-signal paradigm. Trends Cogn Sci. (2008) 12:418–24. 10.1016/j.tics.2008.07.005 18799345 PMC2709177

[13] Verbruggen F Logan GD. Models of response inhibition in the stop-signal and stop-change paradigms. Neurosci Biobehav Rev. (2009) 33:647–61. 10.1016/j.neubiorev.2008.08.014 18822313 PMC2696813

[14] Logan GD Cowan WB Davis KA. On the ability to inhibit simple and choice reaction time responses: a model and a method. J Exp Psychol Hum Percept Perform. (1984) 10:276–91. 10.1037/0096-1523.10.2.276 6232345

[15] Logan GD Van Zandt T Verbruggen F Wagenmakers EJ. On the ability to inhibit thought and action: general and special theories of an act of control. Psychol Rev. (2014) 121:66–95. 10.1037/a0035230 24490789

[16] Aron AR Poldrack RA. The cognitive neuroscience of response inhibition: relevance for genetic research in attention-deficit/hyperactivity disorder. Biol Psychiatry. (2005) 57:1285–92. 10.1016/j.biopsych.2004.10.026 15950000

[17] Lipszyc J Schachar R. Inhibitory control and psychopathology: a meta-analysis of studies using the stop signal task. J Int Neuropsychol Soc. (2010) 16:1064–76. 10.1017/S1355617710000895 20719043

[18] Bellgrove MA Chambers CD Vance A Hall N Karamitsios M Bradshaw JL. Lateralized deficit of response inhibition in early-onset schizophrenia. Psychol Med. (2006) 36:495–505. 10.1017/S0033291705006409 16336744

[19] Derntl B Habel U. Angry but not neutral faces facilitate response inhibition in schizophrenia patients. Eur Arch Psychiatry Clin Neurosci. (2017) 267:621–7. 10.1007/s00406-016-0748-8 27866272

[20] Egashira K Matsuo K Nakashima M Watanuki T Harada K Nakano M. Blunted brain activation in patients with schizophrenia in response to emotional cognitive inhibition: a functional near-infrared spectroscopy study. Schizophr Res. (2015) 162:196–204. 10.1016/j.schres.2014.12.038 25595654

[21] Enticott PG Ogloff JR Bradshaw JL. Response inhibition and impulsivity in schizophrenia. Psychiatry Res. (2008) 157:251–4. 10.1016/j.psychres.2007.04.007 17916385

[22] Vercammen A Morris R Green MJ Lenroot R Kulkarni J Carr VJ. Reduced neural activity of the prefrontal cognitive control circuitry during response inhibition to negative words in people with schizophrenia. J Psychiatry Neurosci. (2012) 37:379–88. 10.1503/jpn.110088 22617625 PMC3493093

[23] Zheng Q Yang TX Ye Z. Emotional stop cues facilitate inhibitory control in schizophrenia. J Int Neuropsychol Soc. (2020) 26:286–93. 10.1017/S1355617719001152 31694734

[24] Chamberlain SR Fineberg NA Blackwell AD Robbins TW Sahakian BJ. Motor inhibition and cognitive flexibility in obsessive-compulsive disorder and trichotillomania. Am J Psychiatry. (2006) 163:1282–4. 10.1176/ajp.2006.163.7.1282 16816237

[25] Aker M Bo R Harmer C Stiles TC Landro NI. Inhibition and response to error in remitted major depression. Psychiatry Res. (2016) 235:116–22. 10.1016/j.psychres.2015.11.038 26639650

[26] Langenecker SA Kennedy SE Guidotti LM Briceno EM Own LS Hooven T. Frontal and limbic activation during inhibitory control predicts treatment response in major depressive disorder. Biol Psychiatry. (2007) 62:1272–80. 10.1016/j.biopsych.2007.02.019 17585888 PMC2860742

[27] Li FF Chen XL Zhang YT Li RT Li X. The role of prepotent response inhibition and interference control in depression. Cogn Neuropsychiatry. (2021) 26:441–54. 10.1080/13546805.2021.1987872 34617501

[28] Svaldi J Naumann E Trentowska M Schmitz F. General and food-specific inhibitory deficits in binge eating disorder. Int J Eat Disord. (2014) 47:534–42. 10.1002/eat.22260 24573740

[29] Herbert C Sütterlin S. Response inhibition and memory retrieval of emotional target words: evidence from an emotional stop-signal task. J Behav Brain Sci. (2011) 01:153–9. 10.4236/jbbs.2011.13020

[30] Rebetez MM Rochat L Billieux J Gay P Van der Linden M. Do emotional stimuli interfere with two distinct components of inhibition? Cogn Emot. (2015) 29:559–67. 10.1080/02699931.2014.922054 24885111

[31] Krypotos AM Jahfari S van Ast VA Kindt M Forstmann BU. Individual differences in heart rate variability predict the degree of slowing during response inhibition and initiation in the presence of emotional stimuli. Front Psychol. (2011) 2:278. 10.3389/fpsyg.2011.00278 22059080 PMC3204574

[32] Verbruggen F De Houwer J. Do emotional stimuli interfere with response inhibition? Evidence from the stop signal paradigm. Cognit Emot. (2007) 21:391–403. 10.1080/0269993060062508134530318

[33] Öhman A Flykt A Esteves F. Emotion drives attention: detecting the snake in the grass. J Exp Psychol Gen. (2001) 130:466–78. 10.1037/0096-3445.130.3.466 11561921

[34] Habel U Chechko N Pauly K Koch K Backes V Seiferth N. Neural correlates of emotion recognition in schizophrenia. Schizophr Res. (2010) 122:113–23. 10.1016/j.schres.2010.06.009 20663646

[35] Phillips LK Seidman LJ. Emotion processing in persons at risk for schizophrenia. Schizophr Bull. (2008) 34:888–903. 10.1093/schbul/sbn085 18644853 PMC2518637

[36] Pinkham AE Brensinger C Kohler C Gur RE Gur RC. Actively paranoid patients with schizophrenia over attribute anger to neutral faces. Schizophr Res. (2011) 125:174–8. 10.1016/j.schres.2010.11.006 21112186 PMC3031724

[37] Seiferth NY Pauly K Kellermann T Shah NJ Ott G Herpertz-Dahlmann B. Neuronal correlates of facial emotion discrimination in early onset schizophrenia. Neuropsychopharmacology. (2009) 34:477–87. 10.1038/npp.2008.93 18580874

[38] Liang JN Hu WT Gu YT Cheng TH Geng JS Wang KL. Impairment of response inhibition to emotional face stimuli in individuals with subclinical depression. Psych J. (2022) 11:327–34. 10.1002/pchj.548 35419989

[39] Camfield DA Burton TK De Blasio FM Barry RJ Croft RJ. Erp components associated with an indirect emotional stop signal task in healthy and depressed participants. Int J Psychophysiol. (2018) 124:12–25. 10.1016/j.ijpsycho.2017.12.008 29278691

[40] Yang H Liu Q Peng W Liu Z Chu J Zheng K. Impaired impulse inhibition of emotional stimuli in patients with borderline personality disorder. Sci Rep. (2021) 11:16628. 10.1038/s41598-021-96166-1 34404887 PMC8371102

[41] Auerbach RP Stewart JG Stanton CH Mueller EM Pizzagalli DA. Emotion-processing biases and resting eeg activity in depressed adolescents. Depress Anxiety. (2015) 32:693–701. 10.1002/da.22381 26032684 PMC4558362

[42] Bourke C Douglas K Porter R. Processing of facial emotion expression in major depression: a review. Aust New Zeal J Psychiatry. (2010) 44:681–96. 10.3109/00048674.2010.496359 20636189

[43] Gur RC Erwin RJ Gur RE Zwil AS Heimberg C Kraemer HC. Facial emotion discrimination: II. Behavioral findings in depression. Psychiatry Res. (1992) 42:241–51. 10.1016/0165-1781(92)90116-K 1496056

[44] Lee L Harkness KL Sabbagh MA Jacobson JA. Mental state decoding abilities in clinical depression. J Affect Disord. (2005) 86:247–58. 10.1016/j.jad.2005.02.007 15935244

[45] Leppanen JM Milders M Bell JS Terriere E Hietanen JK. Depression biases the recognition of emotionally neutral faces. Psychiatry Res. (2004) 128:123–33. 10.1016/j.psychres.2004.05.020 15488955

[46] Rubinow DR Post RM. Impaired recognition of affect in facial expression in depressed patients. Biol Psychiatry. (1992) 31:947–53. 10.1016/0006-3223(92)90120-O 1637932

[47] Bouhuys AL Geerts E Gordijn MCM. Depressed patients' perceptions of facial emotions in depressed and remitted states are associated with relapse: a longitudinal study. J Nerv Ment Dis. (1999) 187:595–602. 10.1097/00005053-199910000-00002 10535652

[48] Bouhuys AL Geerts E Mersch PP Jenner JA. Nonverbal interpersonal sensitivity and persistence of depression: perception of emotions in schematic faces. Psychiatry Res. (1996) 64:193–203. 10.1016/S0165-1781(96)02930-7 8944397

[49] Yoon KL Zinbarg RE. Threat is in the eye of the beholder: social anxiety and the interpretation of ambiguous facial expressions. Behav Res Ther. (2007) 45:839–47. 10.1016/j.brat.2006.05.004 16797485

[50] Couette M Mouchabac S Bourla A Nuss P Ferreri F. Social cognition in post-traumatic stress disorder: a systematic review. Br J Clin Psychol. (2020) 59:117–38. 10.1111/bjc.12238 31696974

[51] Heyn SA Schmit C Keding TJ Wolf R Herringa RJ. Neurobehavioral correlates of impaired emotion recognition in pediatric PTSD. Dev Psychopathol. (2022) 34:946–56. 10.1017/S0954579420001704 33487187 PMC9717496

[52] Ozturk A Kilic A Deveci E Kirpinar I. Investigation of facial emotion recognition, alexithymia, and levels of anxiety and depression in patients with somatic symptoms and related disorders. Neuropsychiatr Dis Treat. (2016) 12:1047–53. 10.2147/NDT.S106989 27199559 PMC4857827

[53] Pedrosa Gil F Ridout N Kessler H Neuffer M Schoechlin C Traue HC. Facial emotion recognition and alexithymia in adults with somatoform disorders. Depress Anxiety. (2008) 25:E133–41. 10.1002/da.20440 18033726

[54] Robbins TW Gillan CM Smith DG de Wit S Ersche KD. Neurocognitive endophenotypes of impulsivity and compulsivity: towards dimensional psychiatry. Trends Cogn Sci. (2012) 16:81–91. 10.1016/j.tics.2011.11.009 22155014

[55] Whiteside SP Lynam DR. The five factor model and impulsivity: using a structural model of personality to understand impulsivity. Pers Individ Dif. (2001) 30:669–89. 10.1016/S0191-8869(00)00064-7

[56] Cyders MA Coskunpinar A. Measurement of constructs using self-report and behavioral lab tasks: is there overlap in nomothetic span and construct representation for impulsivity? Clin Psychol Rev. (2011) 31:965–82. 10.1016/j.cpr.2011.06.001 21733491

[57] d'Acremont M Linden MV. Adolescent impulsivity: findings from a community sample. J Youth Adolesc. (2005) 34:427–35. 10.1007/s10964-005-7260-1

[58] Whiteside SP Lynam DR Miller JD Reynolds SK. Validation of the upps impulsive behaviour scale: a four-factor model of impulsivity. Eur J Pers. (2020) 19:559–74. 10.1002/per.556

[59] Wilbertz T Deserno L Horstmann A Neumann J Villringer A Heinze HJ. Response inhibition and its relation to multidimensional impulsivity. Neuroimage. (2014) 103:241–8. 10.1016/j.neuroimage.2014.09.021 25241087

[60] Gay P Rochat L Billieux J d'Acremont M Van der Linden M. Heterogeneous inhibition processes involved in different facets of self-reported impulsivity: evidence from a community sample. Acta Psychol. (2008) 129:332–9. 10.1016/j.actpsy.2008.08.010 18851842

[61] Roxburgh AD White DJ Cornwell BR. Negative urgency is related to impaired response inhibition during threatening conditions. Acta Psychol (Amst). (2022) 228:103648. 10.1016/j.actpsy.2022.103648 35777308

[62] Gabbard GO. Psychotherapy in psychiatry. Int Rev Psychiatry. (2007) 19:5–12. 10.1080/09540260601080813 17365154

[63] Viamontes G Beitman B. Neural substrates of psychotherapeutic change part ii: beyond default mode. Psychiatr Ann. (2006) 36:238–46. 10.3928/00485713-20060401-04

[64] Congdon E Mumford JA Cohen JR Galvan A Canli T Poldrack RA. Measurement and reliability of response inhibition. Front Psychol. (2012) 3:37. 10.3389/fpsyg.2012.00037 22363308 PMC3283117

[65] Gur RC Sara R Hagendoorn M Marom O Hughett P Macy L. A method for obtaining 3-dimensional facial expressions and its standardization for use in neurocognitive studies. J Neurosci Methods. (2002) 115:137–43. 10.1016/S0165-0270(02)00006-7 11992665

[66] Li CS Huang C Constable RT Sinha R. Imaging response inhibition in a stop-signal task: neural correlates independent of signal monitoring and post-response processing. J Neurosci. (2006) 26:186–92. 10.1523/JNEUROSCI.3741-05.2006 16399686 PMC6674298

[67] Li CS Yan P Sinha R Lee TW. Subcortical processes of motor response inhibition during a stop signal task. Neuroimage. (2008) 41:1352–63. 10.1016/j.neuroimage.2008.04.023 18485743 PMC2474693

[68] Li CS Zhang S Duann JR Yan P Sinha R Mazure CM. Gender differences in cognitive control: an extended investigation of the stop signal task. Brain Imaging Behav. (2009) 3:262–76. 10.1007/s11682-009-9068-1 19701485 PMC2728908

[69] Pawliczek CM Derntl B Kellermann T Kohn N Gur RC Habel U. Inhibitory control and trait aggression: neural and behavioral insights using the emotional stop signal task. Neuroimage. (2013) 79:264–74. 10.1016/j.neuroimage.2013.04.104 23660028

[70] Schmidt RE Gay P d'Acremont M Van der Linden M. A German adaptation of the upps impulsive behavior scale: psychometric properties and factor structure. Swiss J Psychol. (2008) 67:107–12. 10.1024/1421-0185.67.2.107

[71] Gräfe K Zipfel S Herzog W Löwe B. Screening Psychischer Störungen Mit Dem “Gesundheitsfragebogen Für Patienten (Phq-D)”. Diagnostica. (2004) 50:171–81. 10.1026/0012-1924.50.4.171

[72] Muhlberg C Mathar D Villringer A Horstmann A Neumann J. Stopping at the sight of food - how gender and obesity impact on response inhibition. Appetite. (2016) 107:663–76. 10.1016/j.appet.2016.08.121 27592420

[73] Marwood L Wise T Perkins AM Cleare AJ. Meta-analyses of the neural mechanisms and predictors of response to psychotherapy in depression and anxiety. Neurosci Biobehav Rev. (2018) 95:61–72. 10.1016/j.neubiorev.2018.09.022 30278195 PMC6267850

[74] Goldapple K Segal Z Garson C Lau M Bieling P Kennedy S. Modulation of cortical-limbic pathways in major depression: treatment-specific effects of cognitive behavior therapy. Arch Gen Psychiatry. (2004) 61:34–41. 10.1001/archpsyc.61.1.34 14706942

[75] Beutel ME Stark R Pan H Silbersweig D Dietrich S. Changes of brain activation pre- post short-term psychodynamic inpatient psychotherapy: an fmri study of panic disorder patients. Psychiatry Res. (2010) 184:96–104. 10.1016/j.pscychresns.2010.06.005 20933374

[76] Holzel BK Hoge EA Greve DN Gard T Creswell JD Brown KW. Neural mechanisms of symptom improvements in generalized anxiety disorder following mindfulness training. Neuroimage Clin. (2013) 2:448–58. 10.1016/j.nicl.2013.03.011 24179799 PMC3777795

[77] Allen KJD Johnson SL Burke TA Sammon MM Wu C Kramer MA. Validation of an emotional stop-signal task to probe individual differences in emotional response inhibition: relationships with positive and negative urgency. Brain Neurosci Adv. (2021) 5:23982128211058269. 10.1177/23982128211058269 34841088 PMC8619735

[78] Biringer E Lundervold A Stordal K Mykletun A Egeland J Bottlender R. executive function improvement upon remission of recurrent unipolar depression. Eur Arch Psychiatry Clin Neurosci. (2005) 255:373–80. 10.1007/s00406-005-0577-7 15793669

[79] Lahr D Beblo T Hartje W. Cognitive performance and subjective complaints before and after remission of major depression. Cogn Neuropsychiatry. (2007) 12:25–45. 10.1080/13546800600714791 17162445

[80] Hammar Å Lund A Hugdahl K. Long-lasting cognitive impairment in unipolar major depression: a 6-month follow-up study. Psychiatry Res. (2003) 118:189–96. 10.1016/S0165-1781(03)00075-1 12798984

[81] Neu P Bajbouj M Schilling A Godemann F Berman RM Schlattmann P. Cognitive function over the treatment course of depression in middle-aged patients: correlation with brain MRI signal hyperintensities. J Psychiatr Res. (2005) 39:129–35. 10.1016/j.jpsychires.2004.06.004 15589560

[82] Weiland-Fiedler P Erickson K Waldeck T Luckenbaugh DA Pike D Bonne O. Evidence for continuing neuropsychological impairments in depression. J Affect Disord. (2004) 82:253–8. 10.1016/j.jad.2003.10.009 15488254

[83] Leotti LA Wager TD. Motivational influences on response inhibition measures. J Exp Psychol Hum Percept Perform. (2010) 36:430–47. 10.1037/a0016802 20364928 PMC3983778

[84] Andrade LH Alonso J Mneimneh Z Wells JE Al-Hamzawi A Borges G. Barriers to mental health treatment: results from the who world mental health surveys. Psychol Med. (2014) 44:1303–17. 10.1017/S0033291713001943 23931656 PMC4100460

[85] Clery-Melin ML Jollant F Gorwood P. Reward systems and cognitions in major depressive disorder. CNS Spectr. (2019) 24:64–77. 10.1017/S1092852918001335 30472971

[86] Jardine J Bowman R Doherty G. Digital interventions to enhance readiness for psychological therapy: scoping review. J Med Internet Res. (2022) 24:e37851. 10.2196/37851 36040782 PMC9472056

[87] Koetsier GC Volkers AC Tulen JH Passchier J van den Broek WW Bruijn JA. Cpt performance in major depressive disorder before and after treatment with imipramine or fluvoxamine. J Psychiatr Res. (2002) 36:391–7. 10.1016/S0022-3956(02)00026-2 12393308

[88] World Health Organization. International Statistical Classification of Diseases and Related Health Problems. 10th ed. (2016). Available online at: https://icd.who.int/browse10/2016/en

