# Supplementary material for: The impact of emotional stimuli on response inhibition in an inpatient and day-hospital patient psychosomatic cohort
Source: Front Psychiatry. 2023 Jun 30;14:1176721. doi: 10.3389/fpsyt.2023.1176721 (PMC10347415; doi:10.3389/fpsyt.2023.1176721)
Supplement: Supplementary file 1 [file Data_Sheet_1.docx]

Supplementary Material

The impact of emotional stimuli on response inhibition in an inpatient psychosomatic cohort

Sina Westbomke, Kathrin Schag, Birgit Derntl, Stephan Zipfel, Andreas Stengel^*^

*** Correspondence:** Andreas Stengel, Andreas.stengel@med.uni-tuebingen.de

# Supplementary Figures and Tables

**Supplementary Table 1** Performance of the whole group in the emotional stop-signal

task (n=101)

| **Parameter** | **T0** | **p_e_** | **T1** | **p_e_** | **p_t_** |
| --- | --- | --- | --- | --- | --- |
| Accuracy of go trials (%) | | | | | |
| Total | 91.41% |  | 91.87% |  | .555 |
| Anger | 91.97% |  | 91.72% |  | .765 |
| Neutral | 90.89% | **.060** | 91.85% | .824 | .291 |
| RTmed (ms, mean ± SD) | | | | | |
| Anger | 636.18 (±12.3) |  | 636.05 (±20.45) |  | .994 |
| Neutral | 636.6 (±12.27) | 1.00 | 640.39 (±20.12) | .296 | .823 |

Intention to treat analysis of the data; RT_med_, median reactiont in go trials; SSD, stop-

signal delay; SSRT, stop-signal reaction time T0, baseline measurement; T1

discharge measurement. p_e_ p emotion compared_,_ p_t_ p time compared; Significant p

values are displayed in bold.

**Supplementary Table 2** Performance in the emotional stop signal task according to diagnoses

| **Parameter** | **Depressive disorders** (n=28) | | | | | **Somatoform disorders** (n=13) | | | **Eating disorders**  (n=3) | | **Anxiety and trauma** (n=13) | |
| --- | --- | --- | --- | --- | --- | --- | --- | --- | --- | --- | --- | --- |
|  | T0 | p_e_ | T1 | p_e_ | p_t_ | T0 | T1 | T0 | | T1 | T0 | T1 |
| Accuracy of go trials (%) | | | | | | | | | | | | |
| Total | 93.18% |  | 93.54% |  | .697 | 93.15% | 92.92% | 93.3% | | 92.3% | 92.62% | 93% |
| Anger | 93.61% |  | 93.68% |  | .928 | 92.31% | 91.85% | 93.67% | | 90.33% | 92.62% | 93.46% |
| Neutral | 92.89% | .143 | 93.21% | .438 | .780 | 94.01% | 93.85% | 92% | | 94% | 92.69% | 92.69% |
| Accuracy of stop trials (%) | | | | | | | | | | | | |
| Total | 52.04% |  | 51.57% |  | .108 | 52.08% | 51.31% | 52% | | 52.67% | 51.77% | 51.39% |
| Anger | 50.86% |  | 50.5% |  | .810 | 52.92% | 50.61% | 52% | | 54.67% | 49.39% | 48.31% |
| Neutral | 53.21% | .316 | 52.64% | .401 | .711 | 51.23% | 52% | 52% | | 50.67% | 54.15% | 54.46% |
| SSRT (ms, mean ± SD) | | | | | | | | | | | | |
| Anger | 217.17 (±47.41) |  | 207.55 (±47.39) |  | .328 | 223.22 (±66.6) | 225.83 (±54.5) | 204.17 (±73.6) | | 176.61 (±51.33) | 222.17 (±45.78) | 217.75 (±65.17) |
| Neutral | 225.90 (±44.30) | .153 | 212.03 (±50.71) | .379 | .153 | 238.47 (±66.76) | 233.04 (±49.11) | 207.02 (±60.53) | | 195.8 (±63.01) | 220.79 (±63.01) | 229.49 (±65.50) |
| SSD (ms, mean ± SD) | | | | | | | | | | | | |
| Anger | 402.25 (±136.80) |  | 419.58 (±162.58) |  | .471 | 433.48 (±154.97) | 398.1 (±148.06) | 410.66 (±284.89) | | 462.39 (±246.03) | 414.27 (±141.65) | 395.98 (±171.68) |
| Neutral | 391.53 (±129.59) | **.005** | 414.29 (±159.15) | **.012** | .318 | 418.73 (±148.87) | 391.04 (±143.09) | 404.65 (±278.81) | | 447.37 (±248.88) | 411.09 (±139.47) | 390.78 (±163.15) |
| RT_med_ (ms, mean ± SD) | | | | | | | | | | | | |
| Anger | 619.41 (±123.71) |  | 627.12 (±139.22) |  | .696 | 656.69 (±130.02) | 623.92 (±137.82) | 614.83 (±211.46) | | 639.0 (±194.76) | 636.42 (±124.59) | 613.73 (±130.17) |
| Neutral | 617.43 (±125.60) | .643 | 626.32 (±135.68) | .864 | .656 | 657.19 (±121.76) | 624..08 (±137.53) | 611.67 (±218.31) | | 643.17 (±188.69) | 631.88 (±118.2) | 620.27 (±130.47) |

Data were analyzed per protocol. RT_med_, median reaction time in go trials; SSD, stop signal delay; SSRT, stop signal reaction time

T0, baseline measurement; T1 discharge measurement. p_e_ p emotion compared_,_ p_t_ p time compared; Significant p values are

displayed in bol

**Supplementary table 3:** Used psychopharmaceutic drugs

| **Medication group** | **Used drugs** | **Patients with this medication (absolute, %)** |
| --- | --- | --- |
| **Selective and non-selective monoamine reuptake inhibitors** | sertraline, paroxetine, citalopram, escitalopram, venlafaxine, milnaciprane, bupropion, | 14 (24.6%) |
| **Benzodiazepines including analogues and derivatives** | lorazepam, lormatazepam, zolpidem | 5 (8.8%) |
| **Tri- and Tetracyclics** | opipramol, trimipramine, amitryptiline, mirtazapine | 7 (12.3%) |
| **Antipsychotics** | olanzapine, quetiapine, melperon | 5 (8.8%) |
| **Pain medication (Opiode and non-opioide)** | valoron, morphine, tramadol, hydromorhon, oxycodone, naproxen, celecoxib, xylocaine, novaminsulfone | 7 (12.3%) |
| **Other** | agomelatin, methylphenidat | 3 (5.3%) |
| **No psychophamaceutic medication** |  | 24 (42.1%) |

Used psychopharmaceutic drugs in the PP-Analysis N=57, Missing data from 10 patients
